# Rapid detection of gram-negative antimicrobial resistance determinants directly from positive blood culture broths using a multiplex PCR system

**DOI:** 10.1128/jcm.00384-25

**Published:** 2025-10-21

**Authors:** Stefanie Marxreiter, Jamie Marino, Katrina Callan, Judith Hargrave, Tricia Alston, Kathy Fauntleroy, Amy Robertson, Barry N. Kreiswirth, Liang Chen, Mariana Castanheira, Matthew Hockin, Andrew C. Hemmert, Amy Davis, Michael J. Satlin, Lars F. Westblade

**Affiliations:** 1 bioMérieux, Inc., Salt Lake City, Utah, USA; 2 Department of Pathology and Laboratory Medicine, Weill Cornell Medicine 12295, New York, New York, USA; 3 NewYork-Presbyterian Hospital, Weill Cornell Medical Center, New York, New York, USA; 4 Cornell University 5922, Ithaca, New York, USA; 5 Center for Discovery and Innovation, Hackensack Meridian Health, Nutley, New Jersey, USA; 6 School of Pharmacy and Pharmaceutical Sciences, University of Buffalo 15497, Buffalo, New York, USA; 7 Element Materials Technology (JMI Laboratories), North Liberty, Iowa, USA; 8 Department of Medicine, Weill Cornell Medicine 12295, New York, New York, USA; 9 Department of Pediatrics, Weill Cornell Medicine 12295, New York, New York, USA; National Institute of Allergy and Infectious Diseases Division of Intramural Research, Bethesda, Maryland, USA

**Keywords:** antimicrobial resistance, blood cultures, gram-negative bacteria, multiplex PCR

## Abstract

Currently available rapid blood culture diagnostics detect few gram-negative resistance determinants, limiting their clinical utility. We prospectively evaluated the prototype BIOFIRE FILMARRAY Antimicrobial Resistance (AMR) Panel, a rapid multiplex PCR test that detects 31 AMR genes, on residual positive blood culture broths from patients with gram-negative bacteremia due to five target organisms at a New York City hospital. Predicted antimicrobial resistance based on the AMR Panel was compared to results from broth microdilution testing of bloodstream isolates recovered in culture. A simulated stewardship study assessed opportunities for the optimization of therapy if the AMR Panel results had been available for patient care in real time. We enrolled 148 patients with gram-negative bacteremia (*Escherichia coli, n* = 75; *Klebsiella pneumoniae, n* = 44; *Pseudomonas aeruginosa, n* = 17; *Enterobacter cloacae* complex*, n* = 9; and *Acinetobacter baumannii, n* = 3). The sensitivity of the AMR Panel for predicting antimicrobial resistance was ≥90% for 10/14 antimicrobial agents in *E. coli* and for 10/16 agents in *K. pneumoniae*. Specificity was ≥90% for 15/17 agents in *E. coli* and for all 16 agents in *K. pneumoniae*. Performance for other organisms was poor. For *E. coli* or *K. pneumoniae* bacteremia, use of the AMR Panel could have led to earlier escalation or de-escalation of β-lactam therapy in a majority of patients compared to what actually occurred. This study demonstrates that a rapid multiplex PCR test with a large menu of AMR genes can be applied to positive blood culture broths to rapidly predict resistance to frontline antimicrobial agents in patients with *E. coli* or *K. pneumoniae* bacteremia.

**IMPORTANCE:**

Patients with gram-negative bacteremia require urgent treatment with antimicrobial agents that are effective against their infecting pathogen. However, conventional laboratory work-up of blood cultures takes days to yield results, and during this time, patients may receive ineffective therapies. We evaluated the prototype BIOFIRE FILMARRAY AMR Panel, an assay that detects 31 genes in gram-negative bacteria that confer resistance to β-lactams, fluoroquinolones, and aminoglycosides in approximately 1 hour, directly from positive blood culture broths, and compared these results to antimicrobial susceptibility testing of isolates recovered in culture. We found that the AMR Panel accurately predicted resistance in *Escherichia coli* and *Klebsiella pneumoniae* to most antimicrobials. Moreover, if results from this assay had been used for patient care, there would have been opportunities to optimize antimicrobial prescribing more quickly than using conventional methods. These data demonstrate how novel molecular assays could optimize care for patients with *E. coli* and *K. pneumoniae* bacteremia.

## INTRODUCTION

Patients with sepsis due to gram-negative bacteremia require timely antimicrobial therapy that is effective against their bloodstream pathogen because the risk of mortality rapidly increases with delays in appropriate therapy (1, 2). Unfortunately, antimicrobial resistance to empirical therapies is common, particularly with the emergence of bacteria that produce extended-spectrum β-lactamases (ESBLs) (3, 4). Conventional blood culture diagnostics require an additional 2–3 days after the culture signals positive to yield antimicrobial susceptibility testing (AST) results (5). These delays contribute to worse outcomes in patients with antimicrobial-resistant pathogens due to delays in effective therapy (6, 7). Moreover, patients infected with highly susceptible pathogens may receive 2–3 additional days of unnecessary broad-spectrum therapy, which exacerbates the problem of antimicrobial resistance. Thus, faster identification of antimicrobial-resistant bacteria in blood cultures is needed to improve patient outcomes and antimicrobial stewardship.

Molecular panels that rapidly identify common gram-negative bloodstream pathogens and a limited number of antimicrobial resistance determinants directly from positive blood culture broths have been developed to address this problem (5, 8–10). These panels generally detect the most common carbapenemases and/or a single ESBL gene, *bla*_CTX-M_. However, they do not detect TEM and SHV ESBLs or narrow-spectrum β-lactamase genes (e.g., *bla*_TEM-1_), AmpC β-lactamase genes, or genes that confer resistance to fluoroquinolones or aminoglycosides. This lack of comprehensive information on gram-negative antimicrobial resistance limits the ability of these molecular assays to positively impact patient care.

The prototype BIOFIRE FILMARRY Antimicrobial Resistance (AMR) Panel (bioMérieux, Salt Lake City, UT, USA) improves upon current molecular panels by providing more comprehensive detection of genetic antimicrobial resistance determinants in gram-negative bacteria. In this study, we evaluated the clinical performance of this prototype assay to predict AST results of gram-negative bloodstream pathogens when applied directly to positive blood culture broths. To evaluate the potential clinical impact of the AMR Panel, we also performed a simulated stewardship study to assess potential opportunities for antimicrobial escalation and de-escalation if results from the AMR Panel had been available to patient care teams in real time. The prototype AMR Panel has not been submitted to or cleared by any regulatory agency at the time of writing and is also not available for sale.

## MATERIALS AND METHODS

### Study design

This prospective observational study enrolled patients at NewYork-Presbyterian/Weill Cornell Medical Center with positive blood cultures from which *Escherichia coli*, *Klebsiella pneumoniae*, *Enterobacter cloacae* complex, *Pseudomonas aeruginosa*, or *Acinetobacter baumannii* were identified using the BIOFIRE FILMARRAY Blood Culture Identification (BCID) Panel (bioMérieux) between December 2018 and April 2019. The BCID Panel detects 10 gram-negative bacterial species, one family, and a single gram-negative antimicrobial resistance gene, *bla*_KPC_ (11), and was part of the standard clinical workflow for positive blood cultures during the study. The BCID Panel reported both *K. pneumoniae* and *Klebsiella variicola* as *K. pneumoniae*. Patients and their blood culture specimens were included in the study if there was growth in the aerobic or anaerobic bottle (BD BACTEC, Becton, Dickinson and Company, Franklin Lakes, NJ, USA), only a single pathogen was identified by the BCID Panel, and ≥1.5 mL residual blood culture broth was available. Only the first eligible episode per patient was evaluated, and specimens from which multiple bacterial pathogens were recovered in culture were excluded. Aliquots from positive blood cultures were frozen at −80°C within 24 hours of signaling positive and subsequently evaluated on the AMR Panel. Bacterial isolates recovered from the blood cultures were frozen and underwent AST using reference broth microdilution (BMD) with frozen panels containing 17 antimicrobial agents at JMI Laboratories (North Liberty, IA, USA) (12, 13). This served as the reference comparator for the performance of the AMR Panel.

We reviewed the hospital’s electronic medical record to obtain clinical data for study subjects, including demographics, comorbidities (14), timing of the standard-of-care microbiologic work-up, patient location and clinical status, source of bacteremia, antimicrobial therapies, duration of hospitalization, and mortality.

### AMR Panel testing and interpretation

The AMR Panel requires approximately 5 minutes of hands-on time and performs nucleic acid extraction, nested PCR amplification, and amplicon melt curve analysis in a closed system, yielding results in approximately 1 hour. It includes 47 assays for 31 genes that confer resistance to penicillins, β-lactam/β-lactamase inhibitors, cephalosporins, aztreonam, carbapenems, aminoglycosides, and ciprofloxacin (Table 1). These PCR assays target regions of high homology of AMR genes in stage 1 and nested-stage 2 PCR reactions.

**TABLE 1:** Designation of resistance based on the detection of antimicrobial resistance gene determinants^*a*^^,^^*b*^^,^^*c*^

AMR determinant	Penicillins and β-lactam/β-lactamase inhibitors				Cephalosporins						Monobactam	Carbapenems			Aminoglycosides		Fluoroquinolone
	Ampicillin	Ampicillin-sulbactam	Amoxicillin-clavulanate	Piperacillin-tazobactam	Cefazolin	Cefoxitin	Cefotaxime	Ceftazidime	Ceftriaxone	Cefepime	Aztreonam	Ertapenem	Imipenem	Meropenem	Gentamicin	Tobramycin	Ciprofloxacin
TEM-1	●	○															
TEM-E104K (ESBL)	●	○			●		●	●	●	●	●						
TEM-R164S/H (ESBL)	●	○			●		●	●	●	●	●						
TEM-G238S/E240K (ESBL)	●	○			●		●	●	●	●	●						
SHV-1	●																
SHV-D179G/N (ESBL)	●				●		●	●	●	●	●						
SHV-G238S/E240K (ESBL)	●				●		●	●	●	●	●						
LEN	●																
CTX-M (ESBL)	●	○			●	●	●	●	●	●	●						
KPC (carbapenemase)	●	●	●	●	●	●	●	●	●	●	●	●	●	●			
NDM (carbapenemase)	●	●	●	●	●	●	●	●	●	●		●	●	●			
VIM (carbapenemase)	●	●	●	●	●	●	●	●	●	●		●	●	●			
IMP (carbapenemase)	●	●	●	●	●	●	●	●	●	●		●	●	●			
OXA-1	●	●	●	○						○							
OXA-48 (carbapenemase)	●	●	●	●	●	●						●	●	●			
OXA-23 (carbapenemase)	●	●	●	●	●	●					●	●	●	●			
OXA-24/40 (carbapenemase)	●	●	●	●	●	●						●	●	●			
OXA-58	●	●	●	●	●	●						●	●	●			
CMY-1/MOX (AmpC)	●	●	●	●	●	●	●	●	●		●						
DHA (AmpC)	●	●	●		●	●	●	●			●						
RmtA-E															●	●	
ArmA															●	●	
AAC(3′)-Ia															●		
AAC(3′)-II															●	●	
AAC(6′)-Ib																●	
AAC(6′)-IIa															●	●	
ANT(2″)-I/aadB															●	●	
APH(3′)-VI																	
AAC(6′)-lb-cr																●	●
ParC—S80I																	●
GyrA—S83F/I/L																	●

^*a*^ ● interprets the organism as resistant to the corresponding antimicrobial agent because detection of the gene typically indicates resistance.

^*b*^ ○ interprets the organism as resistant to the corresponding antimicrobial agent, even though detection of the gene only sometimes indicates resistance.

^*c*^ Empty cells indicate the determinant does not typically confer resistance to the antimicrobial agent.

In this study, 200 µL of positive blood culture broth was added to a BIOFIRE FILMARRAY sample filter-injection vial containing 800 µL of BIOFIRE FILMARRAY sample buffer. This was then sealed, inverted, and injected into a hydrated AMR Panel reagent pouch. The loaded reagent pouches were tested on a BIOFIRE Torch system. Results were generated using an endpoint melt curve analysis. Resistance characterization was determined based on the detection of specific AMR markers outlined in Table 1. Resistance conferred by single-nucleotide polymorphism variants was characterized by comparing the cycle threshold values between wild-type and mutant assays.

### AMR Panel performance

For each specimen, we compared the results of the AMR Panel performed on the positive blood culture broth (susceptible or resistant, based on the presence or absence of resistance determinants in Table 1) to AST results from BMD of the isolate recovered in culture. Sensitivity (proportion of specimens with a resistant result on the AMR Panel among specimens that were not susceptible by BMD), specificity (proportion of specimens with a susceptible result on the AMR Panel among specimens that were susceptible by BMD), positive predictive value (PPV) of having a not susceptible isolate by BMD when the AMR Panel reports resistance, and negative predictive value (NPV) of having a susceptible isolate by BMD when the AMR Panel does not report resistance were calculated for each organism for up to 17 antimicrobial agents. These performance characteristics were selected as the primary analysis instead of categorical agreement and error rates because the AMR Panel predicts resistance but is not an AST method. Moreover, the AMR Panel was compared to a reference method, BMD AST of isolates recovered in culture, and thus we believe sensitivity and specificity are more appropriate than positive percent agreement and negative percent agreement. Intermediate results by BMD were grouped with resistant results because there is no intermediate category for the AMR Panel. If multiple isolates of a target organism were recovered in the same culture, the more resistant isolate for each antimicrobial agent was used as the reference standard. As a supplemental analysis, we also calculated very major errors (proportion of specimens with a susceptible result on the AMR Panel among specimens that were resistant by BMD) and major errors (proportion of specimens with a resistant result on the AMR panel among specimens that were susceptible by BMD). Minor errors and categorical agreement could not be calculated because of the lack of an intermediate result on the AMR Panel.

### Simulated stewardship evaluation for bacteremia due to *E. coli* and *K. pneumoniae*

**Fig 1:**
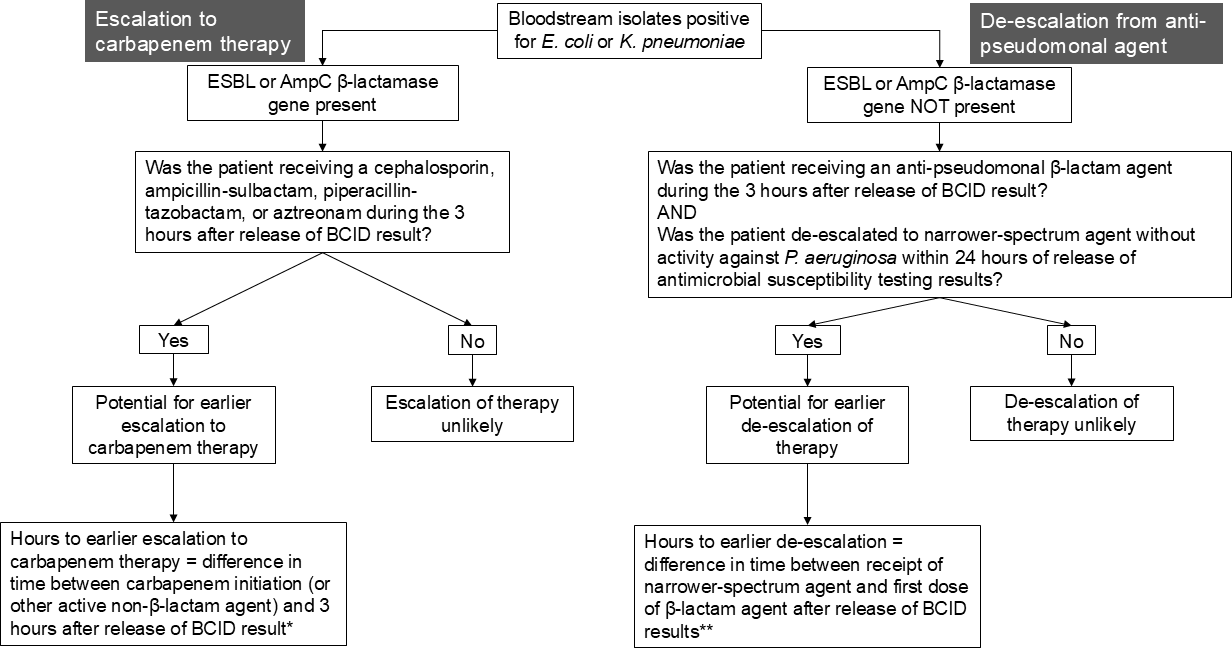
Simulated stewardship algorithm to assess the potential for escalation and de-escalation of antimicrobial therapy based on ESBL and AmpC β-lactamase gene detection if the AMR Panel had been performed immediately after *Escherichia coli* or *Klebsiella pneumoniae* were detected by the BCID Panel. *For escalation of therapy, this algorithm simulates what would have happened if clinicians had acted upon the presence of an ESBL or AmpC β-lactamase gene within 1 hour after the AMR Panel result and compares this to what actually happened. **For de-escalation of therapy, this algorithm simulates what would have happened if clinicians had de-escalated from a broader-spectrum anti-pseudomonal β-lactam agent to a more narrow-spectrum agent at the time the next antimicrobial dose was due after the AMR Panel result, when no ESBL or AmpC β-lactamase gene was detected, and compares this to what actually happened.

Results of the prototype AMR Panel were not reported to patient care teams, and therefore, we could not evaluate the clinical impact of the assay. Instead, we developed an algorithm (Fig. 1) to identify potential opportunities for escalation or de-escalation of antibacterial therapy if the AMR Panel had been performed immediately after the BCID Panel identified gram-negative bacteria, the AMR Panel results had been reported to patient care teams within 2 hours after the BCID Panel result, and clinicians had acted upon this information within 1 hour after receiving AMR Panel results. We focused this analysis on the two most common gram-negative bloodstream isolates, *E. coli* and *K. pneumoniae*. If an ESBL or AmpC β-lactamase gene was present in the blood culture specimen, we evaluated whether patients could have been escalated to carbapenem therapy earlier using results from the AMR Panel compared to what was actually done using only results from the BCID assay (Fig. 1). If an ESBL or AmpC β-lactamase gene was not present, we evaluated whether patients could have been de-escalated from a broader-spectrum anti-pseudomonal β-lactam agent (e.g., piperacillin-tazobactam, cefepime, or a carbapenem) to a more narrow-spectrum agent (e.g., ceftriaxone) earlier.

## RESULTS

### Study cohort

We enrolled 148 unique patients with bacteremia due to a target pathogen. The median patient age was 69 years (interquartile range [IQR] 55–78; Table S1). Forty-six percent of patients were women, 38% were non-White or of unknown race, and 9% were Hispanic/Latino. The most common comorbid illnesses were a hematologic malignancy (22% of patients), diabetes (21%), and a solid tumor (19%). Urinary (45% of patients) and intra-abdominal (24%) sources of bacteremia were most common. The most common antimicrobial therapies targeting gram-negative bacteria that were being administered when the BCID Panel result was released were piperacillin-tazobactam (54% of patients), meropenem (13%), and ceftriaxone (8%). The bacterial strain subsequently isolated in culture was susceptible to the antimicrobial therapy being administered at this time in 74% of patients. Thirteen (9%) patients died within 30 days of blood culture collection.

### Bloodstream isolates and genes detected in blood culture broths

Of the 148 bloodstream isolates, 75 (51%) were *E. coli*, 44 (30%) were *K. pneumoniae*, 9 (6%) were *E. cloacae* complex, 17 (11%) were *P. aeruginosa*, and 3 (2%) were *A. baumannii*. Two different morphologies of the same species were recovered in 11 (7%) of the blood cultures. The proportions of isolates resistant to each antimicrobial by BMD AST are depicted in Table S2. Ceftriaxone resistance was identified in 22 (29%) *E. coli* isolates, 10 (23%) *K. pneumoniae* isolates, six (67%) *E. cloacae* complex isolates, and two (67%) *A. baumannii* isolates. Meropenem resistance was not detected in *E. coli* but was detected in four (9%) *K. pneumoniae* isolates, two (22%) *E. cloacae* complex isolates, two (12%) *P. aeruginosa isolates*, and one (33%) *A. baumannii* isolate.

The AMR Panel detected TEM-1 and SHV-1 in 41 (28%) and 37 (25%) blood culture broths, respectively (Table 2). In total, 27 (18%) broth specimens harbored CTX-M, and four (3%) harbored TEM or SHV ESBLs. AmpC β-lactamases and OXA-1 were identified in five (3%) and 15 (10%) specimens, respectively. Seven (5%) specimens harbored a carbapenemase. Mutations in *gyrA* at codon 83 were the most common fluoroquinolone resistance determinants (*n* = 51, 35%), and *aac(6′)-Ib-cr* was the most common aminoglycoside resistance determinant (*n* = 16, 11%).

**TABLE 2:** Genetic resistance determinants detected in positive blood culture broths using the prototype BIOFIRE FILMARRAY AMR Panel^*f*^^,^^*g*^

AMR determinant	All (*n* = 148)	*E. coli* (*n* = 75)	*K. pneumoniae*^*a*^ (*n* = 44)	*P. aeruginosa* (*n* = 17)	*E. cloacae* complex (*n* = 9)	*A. baumannii* (*n* = 3)
β-lactamases						
Class A β-lactamases						
TEM-1	41 (28%)	30 (40%)	8 (18%)	0	3 (33%)	0
TEM–G238S/E240K^*b*^	1 (1%)	0	1 (2%)	0	0	0
SHV-1	37 (25%)	1 (1%)	36 (82%)	0	0	0
SHV–G238S/E240K^*b*^	3 (2%)	0	2 (5%)	0	1 (11%)	0
LEN	6 (4%)	0	6 (14%)	0	0	0
CTX-M^*b*^	27 (18%)	19 (25%)	8 (18%)	0	0	0
KPC^*c*^	5 (3%)	0	3 (7%)	0	2 (22%)	0
Class B β-lactamases						
NDM^*c*^	1 (1%)	0	1 (2%)	0	0	0
Class C β-lactamases						
CMY/MOX^*d*^	3 (2%)	1 (1%)	0	0	2 (22%)	0
DHA^*d*^	2 (1%)	1 (1%)	1 (2%)	0	0	0
Class D β-lactamases						
OXA-1	15 (10%)	9 (12%)	5 (11%)	0	1 (11%)	0
OXA-23^*c*^	1 (1%)	0	0	0	0	1 (33%)
Aminoglycoside resistance determinants						
AAC(3′)-II	15 (10%)	12 (16%)	3 (7%)	0	0	0
AAC(6′)-Ib	4 (3%)	1 (1%)	3 (7%)	0	0	0
ANT(2″)-I/aadB	1 (1%)	0	0	0	0	1 (33%)
APH(3′)-VI	1 (1%)	0	1 (2%)	0	0	0
AAC(6′)-Ib-cr^*e*^	16 (11%)	9 (12%)	6 (14%)	0	1 (11%)	0
Fluoroquinolone resistance determinants						
ParC-S80I	37 (25%)	27 (36%)	6 (14%)	0	2 (22%)	2 (67%)
GyrA-S83F/I/L	51 (34%)	38 (51%)	7 (16%)	3 (18%)	2 (22%)	1 (33%)

^*a*^ *K. pneumoniae* includes *K. pneumoniae* (*n* = 39) and *Klebsiella variicola* (*n* = 5).

^*b*^ This enzyme is an extended-spectrum β-lactamase.

^*c*^ This enzyme is a carbapenemase.

^*d*^ This enzyme is an AmpC β-lactamase.

^*e*^ This enzyme may also confer resistance to ciprofloxacin.

^*f*^ Values represent number (percentage of total in column).

^*g*^ The following resistance markers were not detected: TEM–E104K, TEM–R164S/H, SHV–D179G/N, VIM, IMP, OXA-48, OXA-24/40, OXA-58, RmtA-E, ArmA, AAC(3′)-Ia, and AAC(6′)-IIa.

### Performance of the prototype AMR Panel

The performance of the AMR Panel to predict AST results of bloodstream isolates varied by species and antimicrobial agent (Table 3). For *E. coli*, sensitivity to detect resistant isolates was ≥90% for most antimicrobial agents, except for amoxicillin-clavulanate (65%), cefazolin (62%), cefoxitin (40%), and ceftazidime (89%). Specificity (detection of susceptible organisms) was ≥90% for all agents except ampicillin-sulbactam (88%) and ciprofloxacin (81%). For *K. pneumoniae*, sensitivity for detecting resistant isolates was ≥90% for most antimicrobial agents, with exceptions of ampicillin-sulbactam (85%), piperacillin-tazobactam (80%), cefazolin (71%), cefoxitin (63%), and ciprofloxacin (83%). Specificity to detect susceptible *K. pneumoniae* was ≥90% for all agents. The NPV for detecting resistance in *E. coli* and *K. pneumoniae* was ≥90% for all agents except cefazolin. Very major errors and major errors are presented in Table 4.

**TABLE 3:** Performance characteristics of the prototype BIOFIRE FILMARRAY AMR Panel applied directly to positive blood culture broths to detect antimicrobial resistance compared to broth microdilution antimicrobial susceptibility testing performed on bloodstream isolates^*e*^^,*f*^

Antimicrobial agent	*E. coli* (*n* = 75)				*K. pneumoniae* (*n* = 44)				*P. aeruginosa* (*n* = 17)				*E. cloacae* complex (*n* = 9)			
	Sens	Spec	PPV	NPV	Sens	Spec	PPV	NPV	Sens	Spec	PPV	NPV	Sens	Spec	PPV	NPV
Ampicillin	98% (44/45)	100% (30/30)	100%	97%												
Amoxicillin-clavulanate	65% (11/17)	100% (58/58)	100%	91%	90% (9/10)	100% (34/34)	100%	97%								
Ampicillin-sulbactam	95% (39/41)	88% (30/34)	91%	94%	85% (11/13)	100% (31/31)	100%	94%								
Piperacillin-tazobactam	90% (9/10)	98% (64/65)	90%	98%	80% (8/10)	100% (34/34)	100%	94%	0% (0/2)	100% (15/15)	N/A	88%	50% (3/6)	67% (2/3)	75%	40%
Cefazolin	62% (21/34)	100% (41/41)	100%	76%	71% (10/14)	97% (29/30)	100%	88%								
Cefoxitin	40% (2/5)	100% (70/70)	100%	96%	63% (5/8)	100% (36/36)	100%	92%								
Cefotaxime	91% (20/22)	98% (52/53)	95%	96%	100% (10/10)	97% (33/34)	91%	100%					80% (4/5)	75% (3/4)	80%	75%
Ceftriaxone	91% (20/22)	100% (53/53)	100%	96%	90% (9/10)	97% (33/34)	90%	97%					83% (5/6)	100% (3/3)	100%	75%
Ceftazidime	89% (17/19)	93% (52/56)	81%	96%	100% (10/10)	97% (33/34)	91%	100%	0% (0/2)	100% (15/15)	N/A	88%	83% (5/6)	100% (3/3)	100%	75%
Cefepime^*a*^	94% (17/18)	95% (54/57)	85%	98%	100% (8/8)	94% (34/36)	80%	100%	N/A	100% (17/17)	N/A	100%	100% (1/1)	75% (6/8)	33%	100%
Aztreonam	90% (19/21)	96% (52/54)	90%	96%	100% (10/10)	97% (33/34)	91%	100%	0% (0/4)	100% (13/13)	N/A	76%	80% (4/5)	75% (3/4)	80%	75%
Ertapenem	N/A^*b*^	100% (75/75)	N/A^*b*^	100%	100% (4/4)	100% (40/40)	100%	100%					67% (2/3)	100% (6/6)	100%	86%
Imipenem	N/A^*b*^	100% (75/75)	N/A^*b*^	100%	100% (4/4)	100% (40/40)	100%	100%	0% (0/3)	100% (14/14)	N/A	82%	67% (2/3)	100% (6/6)	100%	86%
Meropenem	N/A^*b*^	100% (75/75)	N/A^*b*^	100%	100% (4/4)	100% (40/40)	100%	100%	0% (0/2)	100% (15/15)	N/A	88%	100% (2/2)	100% (7/7)	100%	100%
Gentamicin^*c*^	92% (12/13)	100% (62/62)	100%	98%	75% (3/4)	100% (40/40)	100%	98%	0% (0/3)	100% (14/14)	N/A	82%	0% (0/1)	100% (8/8)	N/A	89%
Tobramycin^*d*^	94% (15/16)	100% (59/59)	100%	98%	100% (9/9)	97% (34/35)	90%	100%	0% (0/1)	100% (16/16)	N/A	94%	50% (1/2)	100% (7/7)	100%	88%
Ciprofloxacin	91% (30/33)	81% (34/42)	79%	92%	83% (10/12)	94% (30/32)	83%	94%	60% (3/5)	100% (12/12)	100%	86%	100% (3/3)	100% (6/6)	100%	100%

^*a*^ Susceptible dose-dependent isolates were grouped with susceptible.

^*b*^ There were no carbapenem-resistant *E. coli* isolates.

^*c*^ Results are based on Clinical and Laboratory Standards Institute (CLSI) breakpoints in M100-S29 (13). The sensitivity, specificity, PPV, and NPV using revised 2024 breakpoints (15) are 86% (12/14), 100% (61/61), 100%, and 97%, respectively, for *E. coli.* Performance metrics are unchanged when applying the revised breakpoints for *K. pneumoniae* and *E. cloacae* complex*.* There are no 2024 CLSI breakpoints for gentamicin and *P. aeruginosa*.

^*d*^ Results are based on CLSI breakpoints in M100-S29 (13). The sensitivity, specificity, PPV, and NPV using revised 2024 breakpoints (15) are 100% (10/10), 100% (34/34), 100%, and 100%, respectively, for *K. pneumoniae*; 0% (0/2), 100% (15/15), N/A, and 88% (15/17), respectively, for *P. aeruginosa.* The performance characteristics are unchanged when applying the revised breakpoints for *E. coli* and *E. cloacae* complex.

^*e*^ Boxes shaded in gray represent intrinsic resistance.

^*f*^ N/A, not applicable; Sens, sensitivity; Spec, specificity. Sensitivity: the proportion of specimens where the AMR Panel reported a resistant result among specimens where reference broth microdilution reported a not susceptible (intermediate or resistant) result. Specificity: the proportion of specimens where the AMR Panel did not report a resistant result among specimens where reference broth microdilution reported a susceptible result. PPV: the predictive value of having a not susceptible isolate by reference broth microdilution when the AMR Panel reported a resistant result. NPV: the predictive value of having a susceptible isolate by reference broth microdilution when the AMR Panel does not report a resistant result.

**TABLE 4:** Very major errors and major errors of the prototype BIOFIRE FILMARRAY AMR Panel applied directly to positive blood culture broths to detect antimicrobial resistance compared to broth microdilution antimicrobial susceptibility testing performed on bloodstream isolates^*e*^^,^^*f*^

Antimicrobial agent	*E. coli* (*n* = 75)		*K. pneumoniae* (*n* = 44)		*P. aeruginosa* (*n* = 17)		*E. cloacae* complex (*n* = 9)	
	VME	ME	VME	ME	VME	ME	VME	ME
Ampicillin	2% (1/45)	0% (0/30)						
Amoxicillin-clavulanate	33% (1/3)	0% (0/58)	17% (1/6)	0% (0/34)				
Ampicillin-sulbactam	7% (2/29)	12% (4/34)	0% (0/11)	0% (0/31)				
Piperacillin-tazobactam	25% (1/4)	2% (1/65)	20% (2/10)	0% (0/34)	100% (1/1)	0% (0/15)	0% (0/2)	33% (1/3)
Cefazolin	13% (3/24)	0% (0/41)	9% (1/11)	3% (1/30)				
Cefoxitin	33% (1/3)	0% (0/70)	33% (2/6)	0% (0/36)				
Cefotaxime	9% (2/22)	2% (1/53)	0% (0/10)	3% (1/34)			20% (1/5)	25% (1/4)
Ceftriaxone	5% (1/21)	0% (0/53)	10% (1/10)	3% (1/34)			17% (1/6)	0% (0/3)
Ceftazidime	11% (2/19)	7% (4/56)	0% (0/10)	3% (1/34)	100% (2/2)	0% (0/15)	20% (1/5)	0% (0/3)
Cefepime^*a*^	6% (1/18)	5% (3/57)	0% (0/8)	6% (2/36)	N/A^*b*^	0% (0/17)	0% (0/1)	25% (2/8)
Aztreonam	5% (1/19)	4% (2/54)	0% (0/9)	3% (1/34)	N/A^*b*^	0% (0/13)	20% (1/5)	25% (1/4)
Ertapenem	N/A^*b*^	0% (0/75)	0% (0/4)	0% (0/40)			33% (1/3)	0% (0/6)
Imipenem	N/A^*b*^	0% (0/75)	0% (0/4)	0% (0/40)	100% (2/2)	0% (0/14)	0% (0/1)	0% (0/6)
Meropenem	N/A^*b*^	0% (0/75)	0% (0/4)	0% (0/40)	100% (2/2)	0% (0/15)	0% (0/1)	0% (0/7)
Gentamicin^*c*^	8% (1/13)	0% (0/62)	25% (1/4)	0% (0/40)	100% (1/1)	0% (0/14)	100% (1/1)	0% (8/8)
Tobramycin^*d*^	8% (1/13)	0% (0/59)	0% (0/6)	3% (1/35)	100% (1/1)	0% (0/16)	50% (1/2)	0% (0/7)
Ciprofloxacin	0% (0/28)	21% (9/42)	9% (1/11)	6% (2/32)	0% (0/3)	0% (0/12)	0% (0/3)	0% (0/6)

^*a*^ Susceptible dose-dependent isolates were grouped with susceptible.

^*b*^ There were no resistant isolates to calculate VME.

^*c*^ Results are based on Clinical and Laboratory Standards Institute (CLSI) breakpoints in M100-S29 (13). The VME and ME rates using revised 2024 breakpoints (15) are 8% (1/13) and 0% (0/61), respectively, for *E. coli.* Performance metrics are unchanged when applying the revised breakpoints for *K. pneumoniae* and *E. cloacae* complex*.* There are no 2024 CLSI breakpoints for gentamicin and *P. aeruginosa*.

^*d*^ Results are based on CLSI breakpoints in M100-S29 (13). The VME and ME rates using revised 2024 breakpoints (15) are 0% (0/9) and 0% (0/34), respectively, for *K. pneumoniae*; 100% (1/1) and 0% (0/15), respectively, for *P. aeruginosa.* The performance characteristics are unchanged when applying the revised breakpoints for *E. coli* and *E. cloacae* complex.

^*e*^ ME, major error; N/A, not applicable; VME, very major error. Results are based on Clinical and Laboratory Standards Institute breakpoints in M100-S29 (13). VME: the proportion of specimens where the AMR Panel reported a susceptible result among specimens where reference broth microdilution reported a resistant result. ME: the proportion of specimens where the AMR Panel reported a resistant result among specimens where reference broth microdilution reported a susceptible result.

^*f*^ Boxes shaded in gray represent intrinsic resistance.

The AMR Panel did not detect resistance to most antimicrobial agents in *P. aeruginosa,* and the sensitivity for detecting resistance in *E. cloacae* complex was <85% for most antimicrobial agents (Table 3). The assay detected OXA-23 in the only blood culture broth that harbored carbapenem-resistant *A. baumannii*, but performance characteristics for *A. baumannii* were not calculated due to the small sample size.

### Simulated stewardship

**Fig 2:**
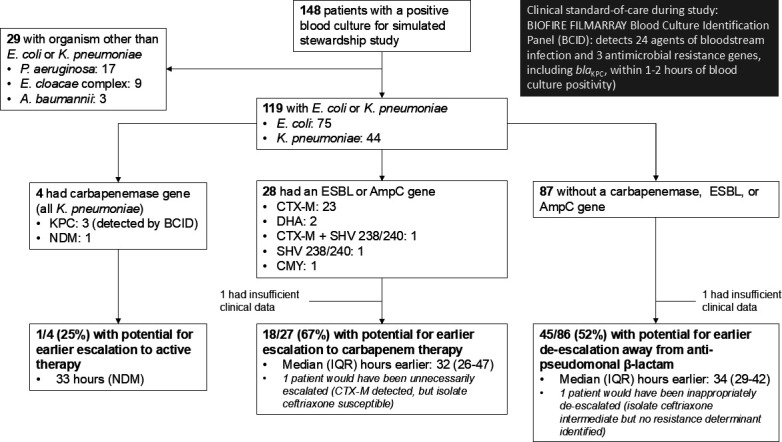
Application of simulated stewardship algorithm to patients with bacteremia due to *Escherichia coli* or *Klebsiella pneumoniae* to determine the frequency with which patients could have received escalation of therapy for bloodstream isolates that harbored a carbapenemase, ESBL, or AmpC β-lactamase gene and de-escalation for bloodstream isolates that did not harbor these genes.

There were 119 patients with bacteremia due to *E. coli* or *K. pneumoniae* who were eligible for the simulated stewardship analysis (Fig. 2). One patient was infected with an NDM-producing *K. pneumoniae,* and if the prototype AMR Panel had been performed after the BCID Panel and acted upon*,* the patient could have been escalated to active therapy 33 hours earlier. Twenty-seven patients had isolates that harbored an ESBL or AmpC β-lactamase and had clinical data available. If the AMR Panel had been performed immediately after the BCID Panel and carbapenem therapy had been administered to patients receiving a cephalosporin, ampicillin-sulbactam, piperacillin-tazobactam, or aztreonam within 1 hour after the release of the AMR Panel results, 18 (67%) of these patients would have been escalated to carbapenem therapy earlier (a median of 32 hours earlier, IQR: 26–47) than what actually occurred. One patient would have been inappropriately escalated to carbapenem therapy because although CTX-M was detected by the AMR Panel, the bloodstream isolate was susceptible to ceftriaxone.

Eighty-six patients were infected with an *E. coli* or *K. pneumoniae* bloodstream isolate that did not harbor a carbapenemase, ESBL, or AmpC β-lactamase and had clinical data available. Forty-five (52%) of these patients were continued on an anti-pseudomonal β-lactam agent after the release of the BCID result and were de-escalated to a narrower-spectrum β-lactam agent within 24 hours after the availability of AST results. Therefore, it is estimated that if the AMR Panel results had been available prior to when the next β-lactam dose was due after the BCID Panel, these patients could have been de-escalated to a narrower-spectrum agent a median of 34 hours (IQR: 29–42) earlier than what actually occurred. One patient could have been inappropriately de-escalated to ceftriaxone because, although the AMR Panel did not detect a ceftriaxone resistance marker, the bloodstream isolate demonstrated intermediate susceptibility to ceftriaxone.

## DISCUSSION

We conducted this study to determine the ability of the AMR Panel, a prototype molecular assay that detects 31 antimicrobial resistance determinants using the BIOFIRE FILMARRAY platform, to predict antimicrobial resistance among five common causes of gram-negative bacteremia when applied directly to positive blood culture broths. We found that sensitivity and specificity for detecting resistance in *E. coli* and *K. pneumoniae* were >90% for most antimicrobial agents, and a “susceptible” result on the AMR Panel (based on lack of detection of resistance determinants) had >90% NPV for resistance to all agents except cefazolin. If the AMR Panel had been available and performed as a reflex test after the BCID Panel results, the majority of patients with *E. coli* and *K. pneumoniae* could have potentially been escalated to carbapenem therapy earlier or de-escalated from an anti-pseudomonal β-lactam earlier, depending on whether ESBL or AmpC β-lactamases were detected. In contrast, the AMR Panel had limited predictive performance for antimicrobial resistance in *P. aeruginosa, E. cloacae* complex, and *A. baumannii*.

The ability of a rapid, highly multiplexed PCR assay like the prototype AMR Panel to be applied directly to positive blood culture broths to predict antimicrobial resistance in *E. coli* and *K. pneumoniae* in approximately 1 hour is important because these pathogens cause two-thirds of gram-negative bacteremias in the United States (16). Current US Food and Drug Administration (FDA)-approved molecular panels for blood cultures, such as the BCID2 Panel, detect carbapenemases and a single ESBL, CTX-M (8–10). The prototype AMR Panel expands upon these targets by also detecting ESBL and narrow-spectrum TEM and SHV β-lactamases, as well as AmpC β-lactamases (e.g., CMY, MOX, and DHA) that confer ceftriaxone resistance. Although CTX-M was the most common ESBL identified, we also identified three CTX-M-negative isolates that were not susceptible to ceftriaxone and had either an ESBL SHV (one *K. pneumoniae* isolate) or an AmpC β-lactamase (one *E. coli* isolate with CMY and one *K. pneumoniae* isolate with DHA). These markers of ceftriaxone resistance would have been missed using current FDA-cleared molecular blood culture panels.

A study of 2,277 *E. coli* and 751 *K*. *pneumoniae* isolates from 56 US hospitals that were carbapenem susceptible but had elevated cephalosporin or aztreonam minimum inhibitory concentration (MIC) values found that 15% and 23% of these *E. coli* and *K. pneumoniae* isolates, respectively, did not harbor CTX-M (17). Among the CTX-M-negative *E. coli* isolates, 67% harbored CMY or DHA and 8% harbored a SHV or TEM ESBL, all targets detected by the prototype AMR Panel. Among the CTX-M-negative *K. pneumoniae* isolates, 78% harbored an SHV ESBL, 16% harbored CMY or DHA, and 2% harbored a TEM ESBL. Detection of these ESBLs and AmpC β-lactamases is clinically important because carbapenems are recommended therapies for bacteremia due to organisms that produce these enzymes, and their detection could lead to earlier appropriate therapy (18, 19). Moreover, given the substantial proportion of CTX-M-negative isolates with elevated cephalosporin MIC values, clinicians may be more likely to rapidly de-escalate from piperacillin-tazobactam, cefepime, or a carbapenem to ceftriaxone if they know the blood culture does not have any of the ESBL or AmpC resistance determinants on the AMR Panel than if they only know it does not have CTX-M.

Notably, the AMR Panel did not reliably detect resistance to cefazolin in both *E. coli* and *K. pneumoniae,* nor resistance to amoxicillin-clavulanate in *E. coli*. Although the reasons for these findings are unclear, this highlights the complicated factors that contribute to resistance to these antimicrobial agents in these organisms. For example, a recent study found that a substantial proportion of *E. coli* and *K. pneumoniae* isolates that do not have an ESBL are susceptible to ceftriaxone but not susceptible to cefazolin (20).

Another potential benefit of a test like the prototype AMR Panel compared to currently used panels is the detection of fluoroquinolone resistance. Although β-lactams are the most common empirical therapies, fluoroquinolones may be used in patients with severe penicillin allergies. Moreover, fluoroquinolones are effective therapies for bacteremia due to urinary tract infections (21, 22), which is the most common source of gram-negative bacteremia (16), and have high oral bioavailability (23), permitting expedited transition from intravenous to oral therapy and potentially earlier hospital discharge (24, 25). In this study, phenotypic ciprofloxacin resistance was not identified by the AMR Panel in 3 of 33 *E. coli* strains and 2 of 12 *K*. *pneumoniae* strains. The AMR Panel detects mutations in *gyrA* at codon 83 and in *parC* at codon 80. While these are the most common mutations that confer ciprofloxacin resistance in Enterobacterales, other *gyrA* and *parC* mutations, efflux pumps, and enzymes that prevent ciprofloxacin from reaching its target may also contribute to ciprofloxacin resistance (26). Further research is needed to better understand additional resistance determinants that would have the highest yield to add to this multiplexed panel to detect ciprofloxacin resistance more comprehensively.

Although the prototype AMR Panel was promising for the detection of resistance in *E. coli* and *K. pneumoniae*, it was less useful for detecting resistance in *P. aeruginosa*, *E. cloacae* complex, and *A. baumannii*. Resistance to β-lactams in *P. aeruginosa* is primarily mediated by mechanisms that are not targets of the AMR Panel, such as pseudomonas-derived cephalosporinases, outer membrane porin modifications, and efflux pumps (27). Members of the *E. cloacae* complex harbor chromosomal AmpC β-lactamases, and resistance to third-generation cephalosporins often occurs due to increased production of these enzymes upon exposure to certain antibiotics (28). These diverse resistance mechanisms create substantial challenges in designing a multiplexed PCR panel to detect resistance in *P. aeruginosa* and *E. cloacae* complex.

This study has limitations. It was a single-center study, and thus, the epidemiology and resistance determinants in gram-negative bloodstream isolates may differ at other institutions. The small number of patients with bacteremia due to *P. aeruginosa*, *E. cloacae* complex, and *A. baumannii* limited the precision of performance estimates for these organisms. The study only evaluated blood cultures with a single gram-negative pathogen detected by BCID. It is unclear how the assay would perform when applied to blood cultures with multiple gram-negative pathogens. Finally, given that the prototype AMR Panel result was not provided to patient care teams, the stewardship component of the study was limited to a simulation. Although it is unknown how physicians would have responded to the information provided by the AMR Panel, particularly outside of regular work hours, it is reasonable to conclude that some patients would have benefited from more appropriate escalation or de-escalation of antimicrobial therapy.

In conclusion, this prospective evaluation of a prototype AMR Panel that detects a broad range of β-lactamases and fluoroquinolone and aminoglycoside resistance determinants found that the assay predicts resistance to many commonly used antimicrobial agents in *E. coli* and *K. pneumoniae*. A simulated stewardship investigation found that results from this assay could have led to faster escalation and de-escalation of antibacterial therapies in many patients. Additional evaluations of an optimized version of this panel are warranted.

## SUPPLEMENTAL MATERIAL

Supplemental Material jcm.00384-25-s0001.docx Tables S1 and S2.

ASM does not own the copyrights to Supplemental Material that may be linked to, or accessed through, an article. The authors have granted ASM a non-exclusive, world-wide license to publish the Supplemental Material files. Please contact the corresponding author directly for reuse.
