# Supplementary material for: Rapid detection of gram-negative antimicrobial resistance determinants directly from positive blood culture broths using a multiplex PCR system
Source: J Clin Microbiol. 2025 Oct 21;63(11):e00384-25. doi: 10.1128/jcm.00384-25 (PMC12607888; doi:10.1128/jcm.00384-25)
Supplement: Supplemental Material — Tables S1 and S2. [file jcm.00384-25-s0001.docx]

**SUPPLEMENTAL MATERIALS**

| Table of Contents Page | 1 |
| --- | --- |
| Supplemental Table 1. Characteristics, treatments, and outcomes of 148 patients with Gram-negative bacteremia in the study cohort | 2-3 |
| Supplemental Table 2. Resistance of bloodstream isolates to antimicrobial agents based on broth microdilution | 4 |

**Supplemental Table 1. Characteristics, treatments, and outcomes of 148 patients with Gram-negative bacteremia in the study cohort**

| **Variable** | **No. (%) or median (IQR)** |
| --- | --- |
| **Demographics** |  |
| Age, years | 69 (55-78) |
| Female gender | 68 (46%) |
| Race |  |
| White | 92 (62%) |
| Black | 30 (20%) |
| Asian | 16 (11%) |
| Other | 5 (3%) |
| Unknown | 5 (3%) |
| Ethnicity |  |
| Not Hispanic or Latino | 131 (89%) |
| Hispanic or Latino | 13 (9%) |
| Unknown | 4 (3%) |
| **Comorbid illnesses** (14) |  |
| Hematologic malignancy | 32 (22%) |
| Diabetes | 31 (21%) |
| Solid malignancy | 28 (19%) |
| Congestive heart failure | 24 (16%) |
| Moderate-to-severe kidney disease | 22 (15%) |
| Cerebrovascular disease | 13 (9%) |
| Dementia | 12 (8%) |
| History of myocardial infarction | 11 (7%) |
| Chronic liver disease | 9 (6%) |
| COPD | 9 (6%) |
| Peripheral vascular disease | 9 (6%) |
| Peptic ulcer disease | 7 (5%) |
| Connective tissue disease | 4 (3%) |
| AIDS | 2 (1%) |
| Charlson Comorbidity Index score | 2 (1-4) |
| **Characteristics at the time of bacteremia onset** |  |
| Patient location |  |
| Outpatient | 7 (5%) |
| Emergency department | 88 (60%) |
| Hospital ward, non-ICU | 39 (26%) |
| ICU | 14 (10%) |
| Neutropenia (absolute neutrophil count ≤500 cells/µL) | 21 (14%) |
| Source of bacteremia |  |
| Urinary tract | 67 (45%) |
| Intra-abdominal (including gut translocation) | 35 (24%) |
| Central venous catheter | 5 (3%) |
| Pulmonary | 5 (3%) |
| Other or unknown | 36 (24%) |
| **Intensive care interventions within 24 hours of blood culture collection** |  |
| ICU care | 41 (28%) |
| Vasopressor support | 25 (17%) |
| Mechanical ventilation | 17 (12%) |
| **Antimicrobial therapies** |  |
| Antimicrobial therapy directed at Gram-negative bacteria at the time the BCID result was released |  |
| Piperacillin-tazobactam | 79 (54%) |
| Meropenem | 19 (13%) |
| Ceftriaxone | 12 (8%) |
| Aztreonam | 6 (4%) |
| Cefepime | 4 (3%) |
| Ceftolozane-tazobactam | 3 (2%) |
| Ertapenem | 2 (1%) |
| Levofloxacin | 2 (1%) |
| Other | 6 (4%) |
| Not on antibacterial therapy | 15 (10%) |
| Receiving active antimicrobial agent vs. Gram-negative bloodstream isolate | 108 (74%) |
| Received active antimicrobial therapy vs. Gram-negative bloodstream isolate at any time | 145 (98%) |
| Received active antimicrobial therapy vs. Gram-negative bloodstream isolate within 24 hours after blood culture collection | 121 (82%) |
| Received active antimicrobial therapy vs. Gram-negative bloodstream isolate within 48 hours after blood culture collection | 133 (90%) |
| **Outcomes** |  |
| Length of hospital stay after bacteremia onset, days | 7 (4-15) |
| 30-day mortality | 13 (9%) |
| 90-day mortality | 21 (14%) |

Abbreviations: AIDS, acquired immunodeficiency syndrome; COPD, chronic obstructive pulmonary disease; ICU, intensive care unit; IQR, interquartile range

**Supplemental Table 2. Resistance of bloodstream isolates to antimicrobial agents based on broth microdilution** (12, 13)

| **Antimicrobial class** | **Antimicrobial agent** | ***E. coli***  **(n=75)** | ***K. pneumoniae*^1^ (n=44)** | ***E. cloacae* complex (n=9)** | ***P. aeruginosa* (n=17)** | ***A. baumannii* (n=3)** |
| --- | --- | --- | --- | --- | --- | --- |
| Penicillins | Ampicillin | 45 (60%) |  |  |  |  |
|  | Amoxicillin-clavulanate | 17 (23%) | 10 (23%) |  |  |  |
|  | Ampicillin-sulbactam | 41 (55%) | 13 (30%) |  |  | 1 (33%) |
|  | Piperacillin-tazobactam | 10 (13%) | 10 (23%) | 6 (67%) | 2 (12%) | 1 (33%) |
| Cephalosporins | Cefazolin | 34 (45%) | 14 (32%) |  |  |  |
|  | Cefoxitin | 5 (7%) | 8 (18%) |  |  |  |
|  | Cefotaxime | 22 (29%) | 10 (23%) | 5 (56%) |  | 1 (33%) |
|  | Ceftriaxone | 22 (29%) | 10 (23%) | 6 (67%) |  | 2 (67%) |
|  | Ceftazidime | 19 (25%) | 10 (23%) | 6 (67%) | 2 (12%) | 0 |
|  | Cefepime | 18 (24%) | 8 (18%) | 1 (11%) | 0 | 1 (33%) |
| Monobactam | Aztreonam | 21 (28%) | 10 (23%) | 5 (56%) | 4 (24%) |  |
| Carbapenems | Ertapenem | 0 | 4 (9%) | 3 (33%) |  |  |
|  | Imipenem | 0 | 4 (9%) | 3 (33%) | 3 (18%) | 1 (33%) |
|  | Meropenem | 0 | 4 (9%) | 2 (22%) | 2 (12%) | 1 (33%) |
| Aminoglycosides | Gentamicin | 13 (17%) | 4 (9%) | 1 (11%) | 3 (18%) | 1 (33%) |
|  | Tobramycin | 16 (21%) | 9 (21%) | 2 (22%) | 1 (6%) | 1 (33%) |
| Fluoroquinolone | Ciprofloxacin | 33 (44%) | 12 (27%) | 3 (33%) | 5 (29%) | 1 (33%) |

Values represent No. (% intermediate or resistant) based on CLSI M100-S29 (13). Boxes shaded in gray represent intrinsic resistance. Isolates that tested intermediate to the antimicrobial agent were considered resistant.

Abbreviations: *A. baumannii*, *Acinetobacter baumannii*; *E. cloacae*, *Enterobacter cloacae*; *E. coli*, *Escherichia coli;* *K. pneumoniae*, *Klebsiella pneumoniae*; *P. aeruginosa*, *Pseudomonas aeruginosa*.

^1^*K. pneumoniae* includes *K. pneumoniae* (n=39) and *Klebsiella variicola* (n=5).
